# “From experts to locals hands” healthcare service planning in sub-Saharan Africa: an insight from the integrated community case management of Ghana

**DOI:** 10.1186/s12913-021-06407-2

**Published:** 2021-04-29

**Authors:** Isaac Annobil, Francis Dakyaga, Mavis Lepiinlia Sillim

**Affiliations:** 1 Ghana Health Service (GHS), District Health Directorate, Ho, Volta Region Ghana; 2 Faculty of Spatial Planning, TU-Dortmund, Dortmund, Germany; 3 Department of Urban and Regional Planning, Ardhi University, Dar es Salaam, Tanzania; 4 Department of Local Governance and City Management, SD Dombo University of Business and Integrated Development Studies, Wa, Ghana

## Abstract

**Background:**

Although community participation remains an essential component globally in healthcare service planning, evidence of how rural communities participate in the planning of rural-based healthcare programs has less been explored in Sub-Saharan Africa.

**Objective:**

We explored communities’ participation in health care planning in hard-to-reach communities, within the context of Integrated Community Case Management (iCCM), a community-based health program implemented in Ghana.

**Methods:**

Qualitative data were collected from eleven (11) hard-to-reach communities through Focus Group Discussions (FGDs), Key Informant Interviews (KIIs) as well as district-level studies (Nadowli-Kaleo, and WA East districts of Ghana). The Rifkin’s spider-gram, framework, for measuring and evaluating community participation in healthcare planning was adapted for the study.

**The results:**

The study found that community participation was superficially conducted by the CHOs. A holistic community needs assessment to create awareness, foster a common understanding of health situations, collaboration, acceptance and ownership of the program were indiscernible. Rather, it took the form of an event, expert-led-definition, devoid of coherence to build locals understanding to gain their support as beneficiaries of the program. Consequently, some of the key requirements of the program, such as resource mobilization by rural residents, Community-based monitoring of the program and the act of leadership towards sustainability of the program were not explicitly found in the beneficiaries’ communities.

**Conclusion and recommendation:**

The study concludes that there is a need to expand the concept of community involvement in iCCM to facilitate communities’ contribution to their healthcare. Also, a transdisciplinary approach is required for engineering and scaling up community-based health programs, empowering VHCs, CBHVs and CHAs to realize success.

## Introduction

Since the era of the Alma Ata Declaration, community participation in health care planning has been an essential component for attaining primary health care [1]. For instance, Article VII of the Alma Ata declaration conference of 1978 showcase the need to utilize the primary healthcare approach to maximize participatory planning of health care services [1]. In the context of social accountability, community participation is pronounced and streamlined in the policies of governments in both low-and-middle-income countries [2, 3]. This evolution is not only influenced by the increasing gap in health staff to patient ratio, but also by the spatial disparities between urban and rural communities with access to health. In this arena, locals are perceived as more knowledgeable of their settings, capable of taking-up affairs, and utilizing healthcare services better when they are at the centre of affairs [3].

Nonetheless, the inherent value of involving the community in health care planning is still an ongoing debate [3–5]. Firstly, it is argued that when community members participate in the recruitment, monitoring, and supervision, it fosters ownership, lessens the cost-of-service delivery, leading to acceptance of the community-based health service [6]. Secondly, the “community is strength” argument, perceives the community as an agent for, diagnosing and prioritizing solutions to health problems confronting their localities [3]. It is perceived to promote broad-based support in the usage of services at the local levels and for fostering cohesiveness towards achieving a common goal [7]. Thirdly, the capabilities point of view perceives the participation of key stakeholders/actors as a prerequisite for holistic acceptance of health programs [3]. Community Health Actors (CHAs) are acknowledged as prime in influencing the participation of community members in healthcare planning [8].

Other factors, advanced on the issues of community participation in health care planning include concern about the recurring resource challenges to promote local participation [4, 9–11]. Rapid appraisal of community health care services planning in Ghana explicitly disclosed how the weak financial support for community participation threatens utilization and sustainability of health program in Ghana [12]. In the case of Kenya, studies found how financial and non-financial factors, including local recognition of Community Health Volunteers (CHV), personal development, and poor working conditions influence the participation of communities in health care planning [4]. However, these contrary observations are less influential in shaping health care paradigms. Given the present pressure on the health systems, and the associated inability to respond adequately to local health concerns [13], the adjunct roles, support of the community health actors (CHAs) maybe supreme for fostering local access to healthcare services in Sub-Saharan Africa [4].

Nonetheless, studies conducted on community participation in health care showcased mixed outcomes across Sub-Saharan Africa. In Tanzania, studies found that health facility committees are useful in assisting the day-to-day operations of clinics [2]. In Ghana, studies on the proficiencies of the Traditional Birth Attendants (TBAs), found that the TBAs have some abilities, which are useful for building partnership with the modern healthcare practitioners towards addressing rural health needs [14]. Others demonstrated how the existing fractions between local healthcare actors and experts, play a role in providing basic healthcare [15].

While the aforementioned studies on community health are useful in theory, a practical assessment of community participation in health care service delivery is less explored. This study aims to explore community participation in health care service delivery, using the case of the Integrated Community Case Management (iCCM) in hard-to-reach communities of Ghana. The iCCM program evolved to complement the CHPS as an equity-based program targeting primarily hard-to-reach communities to improve access to diagnosis and treatment of malaria, pneumonia, and diarrhoea through the Community-Based Health Volunteers (CBHVs). To do so, we used the spider-gram, framework to understand the existing health facilities, the actors involved, the selection of the community-level actors, the conducted of needs assessment, resource mobilization and the act of leadership. The study is advantageous for drawing lessons for health care service planning, programming, and for staging resilient community health care service.

## Health care services planning in sub-Saharan Africa: the rhetoric

### From experts to local hands: healthcare service planning in rural sub-Saharan Africa

Over decades, there has been an increasing debate in health care service planning from the “expert” notion towards a locally driven approach in Sub-Saharan Africa. In this context, Health care planning refers to the continuous process of decision-making, involving both beneficiaries and modern health care practitioners, regarding community health needs, resources mobilization and allocation towards addressing the defined health concerns of the involved communities. It is concerned with the involvement of residents in the conduct of community health needs assessment and its subsequent implementation. The growing interest in local’s participation in health care planning seems a reminiscence of the pre-colonial and traditional health care practices of African society. Besides, it is generating unending conflict between the mainstream modern medical practitioners and that of the local health actors [14]. This longstanding conflict can be deduced from the following; Firstly, the perceived invasion of the roles of one another. Secondly, the perspective that whereas modern health practitioners are better-skilled and equipped with long-term experiences through clinical training, the local actors draw their experiences from cultural orientation [15]. And thirdly, the inability to find a balance of operation between the modern health experts and that of the local health actors.

Haruna and others study of Traditional Birth Attendants in Ghana, attributed the above to earlier efforts made by the World Health Organization (WHO) towards banning the operations of local health actors, particularly, Traditional Birth Attendants (TBAs) and Traditional healers in several rural Sub-Saharan Africa by the opinion of incompetency, unlawful and harmful nature of their operations [14]. The call contributed to limiting the operational activities of local health practitioners and lowered modernist interests for local health services in their respective jurisdictions [14]. However, contemporary challenges in access to health care service including inadequate health facilities, deficient health care service, and the poor road network linking rural communities to the major towns have resulted in some level of involvement of the Traditional Birth Attendants (TBAs), Traditional healers (TH) in health care service delivery [14, 15].

In literature, this evolving trend has either been termed community participation [2, 3, 16] community engagement [8, 17], or community dialogue [18]. Nevertheless, the above terminologies describe the collective involvement of local people in planning and implementing health programs. In conceptual terms, Rifkin argues that there is no standard definition of community and community participation [19]. On that, a useful definition of community participation depends on how community healthcare program objectives are defined [19]. This study specifies community participation as a scale of approaches in engaging community health actors’ (CHA) in the process of the iCCM, planning and implementation, their inputs in decisions that promote iCCM services and sustainability.

Despite the divergence of perspectives postulated on community participation in health care service delivery, participation is commonly likened to improvement in access to health care services and has a direct contribution towards the realization of the Sustainable Development Goals [2, 13]. The iCCM is intrinsically linked to the reduction of infant mortality, and for limiting prevailing health incidences such as malaria, cholera, and typhoid [13] which are key health indicators affecting rural communities in Sub-Saharan Africa.

Health programs, reforms across Sub-Saharan Africa, largely embraced community participation. For instance, in Tanzania, community participation became part and parcel of the health reforms in the 1990s aimed at replacing the centralized with the decentralized system [2]. In other words, a shift from the experts-led-planning towards local initiated planning. This interest culminated in the establishment of community-level structures, including, the Council Health Service Boards (CHSBs) and the health facility committees. In the case of Ghana, the Ministry of Health (MOH) regards community participation as a means to an end [3], and largely as a process whereby individuals, groups and the communities take responsibility for their health and well-being [20]. The focus has been on the community based-healthcare model [21, 22]. In 1977, Ghana adopted the Community-based service delivery model through the Community Health organisations – Community Clinic Attendants and Traditional Birth Attendants influenced by the Alma Ata Declaration in 1978 with a focus on Primary Health Care (PHC) [21]. Through experimentation in 1994, the Community-Based Health Planning and Services (CHPS) program proved successful in delivering essential community-based health services, hence was adopted as a national strategy for improving access, efficiency and quality of healthcare [21].

Since then, packaging and delivering early and appropriate community-based interventions require the utilization of the CHPS program. The CHPS is owned by the government and operated by skilled health officers [22], providing curative and preventive healthcare services [22, 23]. The CHOs provide reproductive, maternal and child health services, manage diarrhoea, treat malaria, acute respiratory infections and childhood illness, comprehensive family planning and childhood immunization outreach in communities [24], and pulling varieties of supports [25–27]. While the iCCM is an equity-based, and a subsidiary program to the CHPS that targets primarily hard-to-reach communities to improve access to diagnosis and treatment of malaria, pneumonia, and diarrhoea through the Community-Based Health Volunteers (CBHVs).

### The context: integrated community case management (iCCM) of Ghana

As indicated earlier, the iCCM program evolved to complement the CHPS as an equity-based program targeting primarily hard-to-reach communities to improve access to diagnosis and treatment of malaria, pneumonia, and diarrhoea through the Community-Based Health Volunteers (CBHVs). It was first piloted in Ghana as Home-Based Care (HBC) for treatment of malaria among children in 1999. However, the revised child health policy strategy in 2009 mandated the CBHVs to administer treatment for malaria (ACT), pneumonia (amoxicillin) and diarrhoea (ORS and zinc tablets) under the iCCM. The Ghana Health Service (GHS) in collaboration with the United Nations Children’s Fund (UNICEF) scaled-up and strengthened the implementation of iCCM across the country, especially in the five (5) northern regions (Northern, Savannah region, North-East region, Upper East and Upper West Regions). The focal aim is to increase access to prompt and effective treatment of children under five years [10, 28].

With the iCCM, Community-Based Health Volunteers (CBHVs) are expected to be trained to deliver basic health support services and essential medicines for children under five. The CBHVs are expected to be accountable to their communities. CHAs such as Village Health Committees (VHCs), religious groups, and youth clubs and the community are expected to provide financial and non-financial support and monitoring of the CBHVs activities [29]. This strategy is perceived as successful when implemented through dialogue with CBHVs, local leaders and using participatory planning processes as the medium [30]. CBHVs do not receive government salary, hence require the support of the community and draw their motivations as selected local health agents of the community [8, 18].

### Analytical approach: community participation in healthcare planning

The study adopts the spider-gram framework, to analyze community participation in healthcare planning in hard-to-reach communities in Northwestern Ghana [16]. The spider-gram framework was propounded by Rifkin and colleagues (1988), for measuring and evaluating community participation in healthcare planning. The framework identified five indicators useful for evaluating community participation in healthcare planning [16]. The indicators include: *Needs assessment, Organization, Management, Resource mobilization* and *Leadership*. The appropriateness and strengths of the spider-gram analytical framework can be deduced from the following; Firstly, it is simple yet useful for characterizing key elements of community participation especially in healthcare planning. Secondly, it is suitable for visualizing and analyzing community participation [31, 32]. For examples, its suitability is endorsed through earlier application in assessing the level of community participation in health programs [31, 32]. This study modifies the variable *‘Organization’* with *CBA selection* and the variable *‘Management’* with *Community driven monitoring* to reflect the relevant aspects of the iCCM program (See Table 1).

**Table 1:** Analytical approach for Community Involvement in health planning

Indicators	Scale on Involvement				
	Very low (+ 1)	Low (+ 2)	Fair (+ 3)	High (+ 4)	Very high (+ 5)
**Needs assessment**	iCCM Identified or imposed by experts	Experts defined; community collects information	Expert defined; Community consulted	The community involved in the decision but played less role to ensure its implementation.	iCCM priorities were identified by the community, they played the lead role in ensuring its existence
**CBHVs selection**	Existing leadership within the communities did not select CBHVs, Imposed by the Health system	Health system-imposed selection criteria and suggested CBHVs for the community.	The Health system engaged the community in determining selection criteria but community leaders did not consult other interest groups	The Health system engaged the community in determining selection criteria but with minimal consultation of all interest groups	The Health system engaged the community in determining selection criteria. Existing leadership within the communities and all other interest groups were consulted for selecting CBHV.
**Community-driven Monitoring**	Monitoring CBHAs activity is never done by community health actors, left solely for the health staff.	Monitoring of CBHAs activity rarely done by CHAs with limited details.	CHAs occasionally Monitor CBHAs activities but with limited detail on how the decision is taken for iCCM.	CHAs regularly monitor CBHVs activities, take management decisions and give feedbacks to CHO.	CHAs engage in regular monitoring of CBAs activities with CHOs, discusses outcomes and support in solving challenges.
**Resource Mobilization**	No form of community support or resource contribution to iCCM.	Community rarely support or contribute to resourcing the iCCM program	Some community mobilization, contribute to resourcing the iCCM program	A considerable amount of resources mobilized by the community to support iCCM activities	A considerable amount of resources raised by the community to support iCCM activities and CHAs have agreed on terms of support to iCCM activities
**Leadership**	CHAs and Health professionals never engaged in consultative decisions on iCCM	CHAs and Health professionals have usual discussions but do not discuss iCCM.	CHAs and Health professionals have usual collaboration and discuss issues concerning iCCM.	CHAs and CHOs have usual consultative meetings with existing community health structures.	A very close link between CHAs and Health professionals, very regular consultative meetings with existing community structures.

*Source:* Adapted from [32, 33]

The n*eeds assessment, CBA selection, Community-driven monitoring, Resource mobilization* and *Leadership* were adapted as the key indicators and defined to resonate with the indicators of Rifkin and others [16]. The process indicators are the *Needs assessment –* the roles played by community members in identifying their health needs towards analyzing, building understanding and commitment of communities, and creating the basis for community inputs and ownership of the program [8, 33]. The *Community-Based Health Volunteer (CBHV) selection* entails the collaborative roles of the health system and the community in engaging local actors for iCCM. And an ongoing process requiring the collaboration of health staffs and communities in recruiting and replacing locals in healthcare services management [34].

The third indicator, *Community-driven monitoring*, involves the participation of the CHAs’ in monitoring iCCM and CBHVs performance at the community level. It is an opportunity for problem-solving and a broader reach of service to local communities [8, 29]. The fourth indicator, *Resource mobilization*, refers to how the community organizes and contributes resources towards iCCM sustainability. And an indication of full community involvement in healthcare planning [35]. The fifth indicator, *Leadership* entails the linkage and collaboration between existing community structures and health professionals in the decision-making. The linkage between communities and the formal health structure gives room for communication and joint problem understanding and solving [8, 36]. Drawing from related studies [32, 33], the study developed a five-point scale ranging from + 1 to + 5, where + 1 depicts a very low involvement, and + 5 depicts very high involvement (see Table 1).

## Methodological approach

### Study areas

**Fig. 1:**
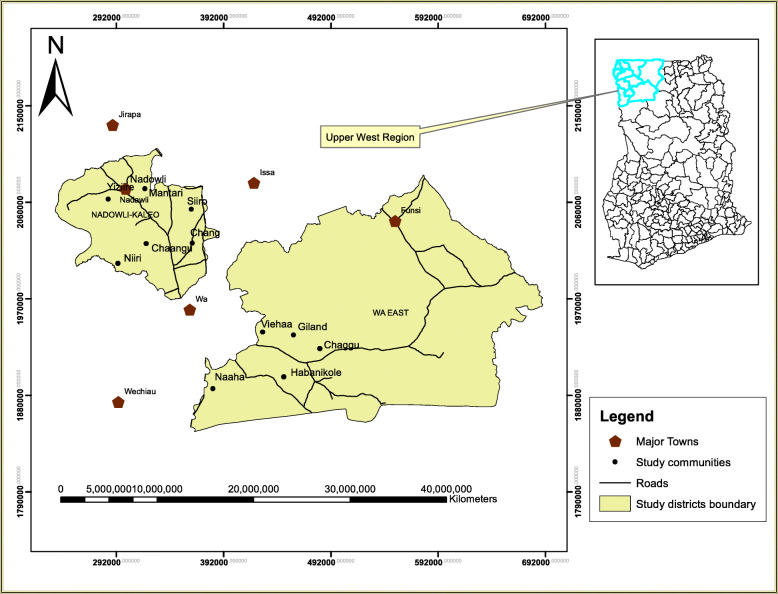
Map of Wa East and Nadowli-Kaleo districts showing the study communities

The study was conducted in 11 hard-to-reach communities (rural communities) in the Wa East and Nadowli-Kaleo districts of the Upper West Region, Ghana, comprising five (5) communities in the Wa East district and six (6) communities in the Nadowli-Kaleo district. The communities included Chang, Chaangu, Chaggu, Giland, Habanikole, Mantari, Naaha, Sirro, Viehaa, Yiziire, and Nirri. These communities were purposively selected due to their hard-to-reach nature. Firstly, they are characterized by dispersed settlement pattern and located far away from the major townships of the districts and the regional capital (Wa), amid very low access to healthcare services [37]. Secondly, both districts record the prevailing incidence of under-five and maternal mortality [38]. Thirdly, both districts were characterized by rural communities and dominated by peasant farmers, who cannot afford the cost of transportation to and from the major health facilities [38]. Fourthly, both districts and the communities have limited health facilities amid inadequate transport modes and poor road networks to major health centres (see Fig. 1).

The poor road network in both districts’ challenges accesses to health. In the Wa East district, over 40% of the roads are less accessible all-year-round [38]. The worse situation occurs between the period of July and September, where most communities in the district get cut-off beyond vehicular reach [37]. Although the state transport, Metro Mass Transit (MMT) is the most reliable means of transport between the regional capital (Wa) and the districts, poor road network challenges all-year-round mobility, especially in the Wa East district [37]. These characteristics necessitated the introduction of the iCCM program.

### Approach and methods

The study employed the qualitative research approach to explore in in-depth, community participation in healthcare planning in hard-to-reach communities in the context of the iCCM [39]. Specifically, the local health actors involved, how they were selected, the needs assessment, locally driven monitoring of the program, resource mobilization and the act of leadership. The qualitative approach was useful in exploring key variables postulated in the Spider-gram, framework on community participation in healthcare service planning [16]. To adequately situate the framework with the result of the study, we reviewed related literature from articles on community participation in healthcare planning in Sub-Saharan Africa [35, 36, 40, 41], and health policy documents, reports and articles in Ghana [3, 12, 14, 25, 33, 38]. We then collected qualitative data on Community health, the actors involved, the conduct of the need’s assessment, the selection of the CBHVs, and how that influenced community-driven monitoring of the process, resources mobilization and leadership for sustainability.

Data were collected through Focus Group Discussion (FGDs) and Key Informant Interviews (KIIs) methods. Twenty (22) Focus Groups Discussions were conducted comprising eleven (11) women and elven (11) men groups with the used of an in-depth interview guide (structured interviews guide). The number of participants per FGDs ranged between 6 and 12 persons and aged between 18 and 70 years. All the participants have lived in the study communities for more than 10 years, as the basis of their rich knowledge and experiences about the iCCM program. Given that the study centred on the general perception and participation of the communities, the face-to-face discussions stimulated debate among the participants and enabled them to come to terms with the realities. However, continuous probing during the FGDs sessions enabled the participants to brainstorm and openly discussed issues about the iCCM program. At the end of each discussion session, the participants were asked to rate their perceived level of participation in the planning of the iCCM in their communities using a 5-point scale (see Table 1), based on the adapted indicators. Each of the interviews’ session lasted approximately, 1 h. 15 min, and the discussions were recorded with audio recorders alongside note-taking.

Key informant interviews were conducted with eleven (11) purposively selected key community-level leaders (Community Health Agents) either the Chiefs, Queen mothers, Assembly members, Clergies, Village health Committee, Care-givers or the Community Association head. Eleven (11) Community-based health Volunteers (CBHVs) were interviewed from each community. The in-depth interview guide was used in the form of a face-to-face conversation. The selection of the key informants was guided by the Community-Health Officers (CHOs) due to their direct interactions with the community local actors under the iCCM program. The selected CBHVs were identified in each community through the effort of the Community Health Agents (CHAs). Data was also collected on the needs assessment, the selection of the CBHV, Community-driven monitoring, resources mobilization and leadership. The interview session for each of the KII lasted between 30 to 50 min.

In the health institutions, Key Informant Interviews (KIIs) were conducted with six (6) purposively sampled health professionals. The District iCCM Coordinators (DICs), the Health Promotion Officers (HPO), of the two districts, in addition to two (2) Community Health Officers (CHOs). The interviews were conducted through face-to-face conversation alongside note-taking and audio recording, by two native speakers of the local dialects (*Dagaare* and *Waala*), also the authors of this paper. The data was validated through community durbars in the respective communities. The consents of the respondents were sought and the interviews recorded with audio recorders for transcription. The results obtained were discussed with the community members for consistency. Indicators that received the highest ranked were discussed and agreed upon as either true reflection or not, whiles conflicting and contradictory views were clarified at the focus group discussion sessions.

The research received ethical clearance and approval from the Ethical Committee of the Graduate School of Tropical Medicine and Global Health, Nagasaki University and the Ghana Health Service Ethical Review Committee. The data was collected between January and March 2018 and validated in February 2019. Due to the remote nature of the sampled communities, three (3) motorbikes were used as the means of transport to the communities by the researchers.

The data recorded were transcribed, reviewed and exported into *Dedoose software* a qualitative software for coding. *Dedoose software* is suitable for analysing data collected in the form of text, photos, audio and video from either qualitative or mixed-method research. In the context of our study, the Dedoose software was used to analyse the text of the qualitative data gathered. The main themes in the sentences were identified, named and the concept given to control the researchers’ subjectivity. All data items were coded and collated with their relevant data excerpts.

## Results and discussions

### Community health – health facilities, proximity and the actors involved in health service delivery

Community-Based Health Planning and Services (CHPS) were the major centres providing basic healthcare services in hard-to-reach communities, though unevenly distributed. Only one community was found with a CHPS. Most communities sought basic healthcare from CHPS in neighbouring communities. The districts health centres and the regional hospital were the major referral centres. Residents disclosed that the distances between communities and the regional and district health centres serve as a challenge to routine healthcare service access, especially to pregnant women and infant mothers. Most of the residents depended on intermediate modes of transport (foot, bicycles, carts), with few utilizing motorbikes and tricycles on hired basis. Communities without CHPS, but with operational motorbikes and tricycles perceived their level of proximity to the nearest health centres as better, compared with communities with less available and operational tricycles and motorbikes (see Table, 2).

These diversities as in spatiality and socio-economic conditions tend to influence the level of reception and acceptability of health programs. As indicated in Table 2, the actors involved in health care service delivery in the study communities comprised individuals and groups. The individual-based actors included the Community-Based Health Volunteer (CBHVs), the Traditional Birth Attendant (TBA), Traditional healer, whilst the group-based actors included the Mother-to-Mother group and the Father-to-Father group. The Mother-to-Mother group is a self-organized group of women concerned with the provision of advice and training of new-born mothers on childcare, hygiene and community health in their community levels. The Father-to-Father group is also a self-organized community-based group of men concerned with the provision of advice to one another on healthcare issues, including the need for husbands to support their wives during pregnancy, by assisting them financially and transporting them to attend ante-natal care when required. Most of the communities were found with functional CBHVs (See Table 2). Though the TBAs were found less active with the advent of the CBHVs, the discussants disclosed that the TBAs – *elderly mothers with nurtured experiences of infant healthcare*, assisted the CBHVs and provided training and support to new-mothers (see Table 2).

**Table 2:**
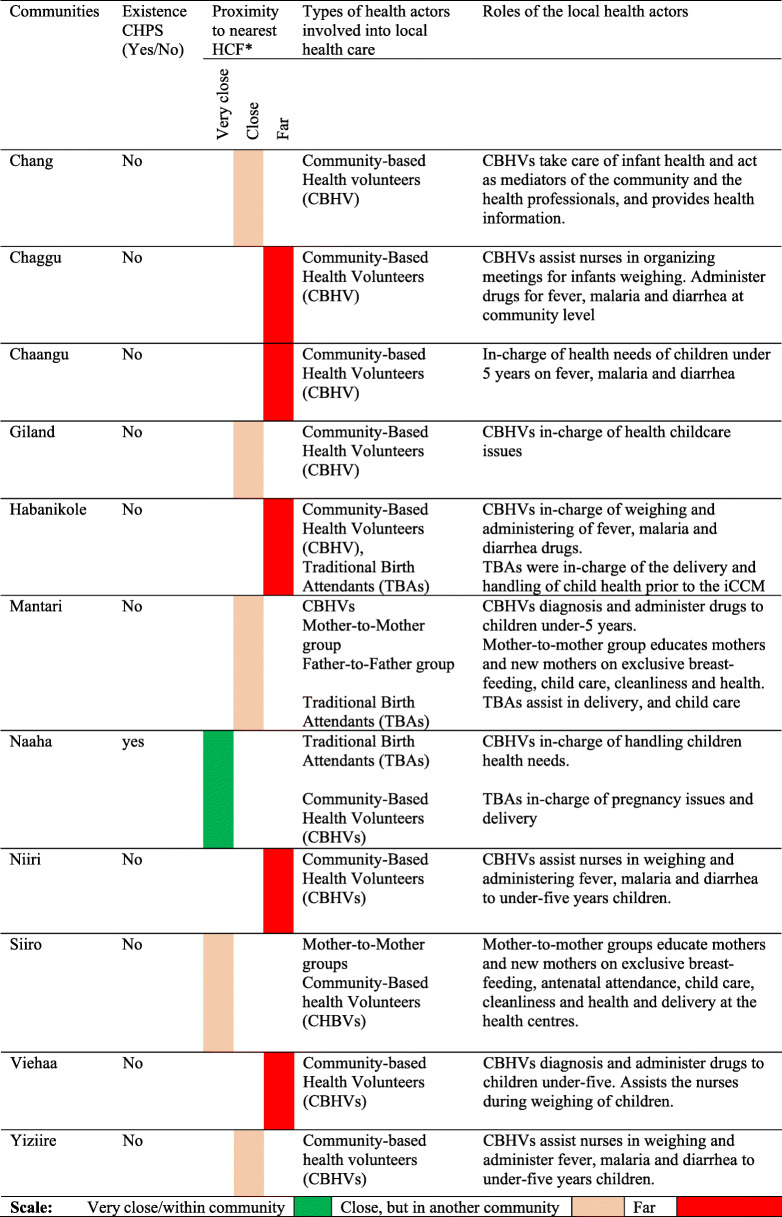
Existing health facilities, proximity, and actors involved

*Source:* Authors’. *HCF – Health Care Facility

### The conduct of the need’s assessment – community health gap identification for intervention

We draw the perspectives of residents in hard-to-reach communities to understand how their health gap was identified towards the introduction of the iCCM program. The study found that the needs assessment was superficially conducted by the Community Health Officers (CHOs), despite its relevance to the sustainability of health’s programs. Consequently, the introduction of the iCCM program was largely shaped by expressed needs and accompanied by perceived needs, where the Community Health Officers provided information to local actors to select Community health volunteers (CBHVs). Accordingly, pregnant women and infant mothers who visited the Community health centres in their neighbouring communities reported prevailing incidences of malaria, fever, diarrhoea, infant mortality, maternal mortality to the CHOs, and were directed to select CBHVs to act as volunteers and mediators of the local health needs and between the residents and the health professionals. Community members selected CBHVs who were later trained on basic local health issues and the administration of drugs; Oral Rehydration Solutions (ORS) against diarrhoea, amoxicillin against pneumonia and (ACT) against malaria among children under 5 years. The case of *Chaang* showcases how expressed needs of some community members culminated in the selection of CHBVs.

“Our children’s health was the issue; children were dying because we have no health centre here. Our children used to fall sick and die commonly either through diarrhoea, cough, malaria or fever, some of the women who went to the Health facility informed the nurses then they asked us to select the volunteers” (FGDs, Men group *Chaang*, 2018).

The Community Health Actors (CHAs) – local leaders, *the Chiefs, Queen mothers, Assembly members, Clergies, Village health Committee, Care-givers or the Community Association heads* who are key in the management of local health programs were not fully involved to enable a common understanding of the purpose. Again, the conduct was not influenced by communal interest but based on perceived and the relative needs of Community Health Officers. In most communities, the conduct took the form of direct selection of the CBHVs;

“…The nurses came and told us to select volunteers, we sat with the chief and elders and selected the volunteers, and the nurses came and praised us for that. The nurses then invited the selected volunteers to *Loggu,* and gave them some training...” (FGD women, *Habanikole,* Wa East district, 2018).

In another case;

“…It was the nurse that came and informed the chief to mobilize the community to select a woman volunteer who is serious and willing to attend to health problems of the community. So together, the chief agreed, and we sat together and they decided that I serve as the volunteer of the community. The entire community agreed to that and since then the nurses give me the drugs…” (CBHV, *Naaha-Zinye,* 2018).

As indicated, community health needs were neither assessed jointly to create awareness and foster collaboration. The locally organized durbars contributed to direct discussion on the selection of Community-based health Volunteer by the locals beneath a new understanding of their health situation.

**Fig. 2:**
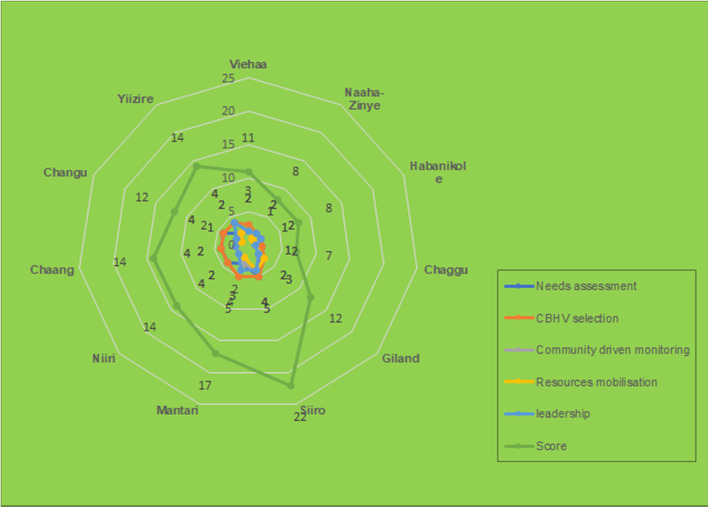
Community participation in healthcare planning

The community needs assessment was participatory conducted in *Yiziire* compared with other communities as shown in Fig. 2**.**

### The selection of community-based-health volunteers (CBHVs) for community health

In most communities, the organization of community-durbars served as the medium of discussion and enables community members to nominate persons, they perceived capable of managing local health affairs. The selection was neither done in the form of appointment nor voting. Rather, capable persons were nominated by the community members during the durbars for collective deliberation and acceptance. The deliberation yielded the acceptance of two-females with perceived capabilities. In *Niiri,* discussants shared the act;

“…We didn’t vote and we sat in the meeting and we mentioned the names of persons we taught were serious and could attend to the health needs of the children and together we also agreed to that…” (FGDs Women & Men, Niiri, Nadowli District, 2018).

In some communities, the CBHVs were proposed by either the chiefs and elders or the Community Health Officers (CHOs) for community acceptance. The chiefs and elders selected women they perceived capable of handling childcare and health and provided information to the community;

“We didn’t vote, the elders of the community met already and decided on the persons to be selected as health volunteers of the community. The chief proposed two-women. After their meeting, they informed the community of the persons who have been selected to serve the health needs of the community” (FGDs with men and Women, Chaggu, 2018).

In *Siiro*, the women group shared a similar view;

“…the CBA’s were selected through the decisions of the following community members; the chief, women group, Queen mother and elders in the presence of the health Officers from the CHPS compounds in a form of a meeting…” (FGDs women, Siiro, Nadowli-Kaleo District, 2018).

However, in the case of *Naaha-Zinye,* the CHOs, selected an elderly woman who they perceived capable of managing local health issues and acceptable by all. The CBHVs were selected from the respective hard-to-reach communities directly and indirectly by members of the communities. Community leaders and caregivers represented the direct beneficiaries of the program. Across the communities, the selection of CBHVs was guided by a set of criteria and attributes - including, persons perceived passionate about community healthcare and humanity, basic literacy, hardworking and willing to accept and work for the community, patient, capable of providing health information to the entire community, positive thinking persons, and passion for Childcare and health. These attributes/criteria were established by the community members in collaboration with the CHOs and perceived worthy for non-paid work. However, participatory selection of community-based health volunteers was found higher in *Siiro* compared with other communities.

### Community-driven monitoring as a basis for sustaining a local health program

We anticipated that participatory driven-needs assessment of community health and selection of the CBHVs could culminate in effective monitoring of the activities of the CBHVs by the Community-health Actors (Local Chiefs and Elders, opinion and religious leaders). In that regard, we perceive this aspect as more of an outcome that can be influenced by the initial stages of health planning. The focal issue here is; *How do community health actors (CHAs) monitor the activities of the Community-Based Health-Volunteers in the hard-to-reach communities?*

The monitoring of the CBHVs activities by the CHAs under the iCCM was indirectly conducted amidst indiscernibility. Only three communities (Mantari, Niiri and Viehaa) were found to have frequently organized community durbars, with the membership of the CHAs as indicated in the Spider-gram framework (Fig. 2). Apart from the aforementioned hard-to-reach communities, meetings organized in other communities neither focus on CBHVs nor tracking their activities. For example, in Changu*. “We hold meetings in the community, but don’t have discussions with the volunteers (CBHVs), we met the last time to discuss the building of the room for drug storage and sometimes meetings with the nurses in the community”* (FGD with women, *Changu*, Wa East district). Similarly, in *Chaang*, monitoring of CBHVs activities only occurs when the CBHVs invite the CHAs or the community for discussions or meetings. As indicated; *“When they invite us, we attend the meeting and when we have durbar, we also invite them. They do invite us to inform us about diseases and how to prevent them…”* (KIIs with CHAs, Chaang, Wa East District, 2018).

The less visibility and indirect regard for monitoring of the CBHVs activities by the CHAs in most hard-to-reach communities are by the following; Firstly, Community-Heath Actors perceived CBHVs as active actors who will work and serve the health needs of their communities irrespective of monitoring. For instance, in Naaha-Zinye, the community perceived the activities of CBHVs as on track despite a lack of monitoring.

“We do not sit for meetings regularly with the CBHVs, it is only when there is the need to make something clear to all the community members. It is people whose work are not going on well that we need to sit and discuss. The volunteers' work has been successful. We don't sit in meetings to discuss anything…” (FGDs Men and Women, Naaha-Zinye, Wa East District, 2018).

Secondly, CHAs are ill-informed and equipped to monitor CBHVs activities. In some communities, the leaders only met based on the information provided by the CHOs on the selection of a Community-Based Health Volunteer. As indicated; *“Not often, the leaders of the community only met them when they were to be selected as CBHVs and after that, they have not met again”* (FGDs women, Chaggu, Wa East district, 2018). Following further probes, the discussants disclosed that lack of knowledge of their roles, clear plan of action and responsibilities in the monitoring of the CBHVs activities serve as the challenge. This can also be deduced from the fact that strategic monitoring of the CBHVs activities required some basic skills and clear indicators of which local actors are less equipped with, but equally not trained to conduct. As justified below; *“Me, the only means I can tell a CBHV is working is just by observing his/her usually activeness in serving the community…*” (KIIs, 2018).

Lastly, community durbars are irregularly organized and less plausible for tracking the progress of the CBHVs activities at the local levels. In the case of Giland, and Habanikole the communities neither organize regular meetings nor meet the CBHVs for discussion on progress. Rather, meetings are organized with CBHVs only when the CHOs visit to discuss health issues with community members.

### Resource mobilization for sustaining operations

Community support in the context of resource mobilization was found limited, except in Viehaa, Siiro*,* and Giland. Locals support for the iCCM program took the form of direct support to the CBHVs livelihoods activities. The support centred on the use of organized groups for farming, weeding and harvesting for the CBHVs and giving due respect for the work of the CBHVs. Except in *Siiro,* the support provided by the residents in Viehaa and Giland targeted the CBHVs as an agent of change. In the case of Viehaa;

“We encourage the CBHV and help them in their farm work and household chores. Sometimes when there is weighing in the community and the CBHVs are engaged with household chores we go and take over the work and allow them to go for the weighing. We also give them the maximum respect by sending our children for medication anytime they are not feeling well” (FGD with women, Viehaa, Wa East District, 2018).

However, community widespread support for the iCCM program was observed only in *Siiro.* Where communal support took the form of improving services and sustaining the iCCM program. The community purchased a tricycle to support the transportation of sick and pregnant women to health centres. Households contributed GHC 15.00 ($ 2.60) monthly to support continuous maintenance and operations of the tricycle.

“The community contributes cash, which comprises of GHC 10.00 ($ 1.71 rate 5.86), from the men and GHC 5 ($ 0.85) from the women. We write down the names of those who pay and give the money to one of the health staffs to be sure that it is in a bank in Wa,^1^ so that anytime we are out of drugs and the support does not come, we can get our own money to buy drugs to support the activities of the CBHV’s. We have executives that go around to take contributions from the various households, the monies are used to assist in the maintenance of the Tricycle and for carrying sick persons to the healthcare centres” (FGDs women and men, Siiro, Nadowli-Kaleo District, 2018).

Though both forms of support are crucial for sustaining the iCCM program, the variation is shaped by community culture of support, perceived individual needs as well as socio-economic conditions in the respective communities. For instance, in most hard-to-reach communities, direct resource mobilisation and support are less forthcoming, but rather in *kind* – respect for the CBHVs work, patronizing their services, availing their children for weighing;

“We pay them in kind and the children they treat also pay them in kind, they are their children. The community respects the activities of the CBHVs and hold their work tightly because we don't have a health centre here. If sickness and disease end in the community, then the activities of the volunteers will also end” (FGDs with men and women, Niiri, Nadowli-Kaleo District, 2018).

In another case;

“We don’t have any help for the CBHV we only benefit from the weighing services and the drugs they administer to the people. Before the start of the iCCM program, we used to contribute to buy drugs just for everyone in the community. But when we started receiving drugs from the Health Centre, we stopped the contribution” (FGDs in Chaang, Chaggu, Changu, Giland, Naaha-Zinye, Niiri*,* 2018).

As indicated, external support fades locals strengthen and initiatives in pursuit of community health. However, the Community Health Actors (CHAs), expressed interest in supporting CBHVs and the program soon., “…*Yes, for the help they deserve, but we cannot knock our chest and provide the kind of help they need. What we think we can do maybe helping them on their farms as we all are farmers, we have not done that any*way…” (FGD, Com-10). However, residents anticipated supporting the work of the CBHVs in the future by mobilising funds at local levels towards purchasing medicines to cater for times of delays delivery, financial support for CBHVs to renew their National Health Insurance Scheme (NHIS), farming for Community-based health Volunteers, means of transport to support intra-community mobility of the CBHVs especially in information dissemination, and medicines administration as kind of motivation for continuous work.

Conclusively, though the capacities of CHAs are well-known in mobilizing and advocating for communal support, in theory, evidence of such capabilities in the context of iCCM was not explicitly found. Communal support for CBHVs, especially in the form of cash mobilization to sustain the purchase of drugs and services, rarely existed. Interviews with CBHVs showcased basic communal neglect of resources support; *“They have not done any cash or material contribution as a community to support the program, they only thank us for the services. There were times that I had to use my motorbike with fuel to take people to the facility or go for drugs”* (CBHVs KII, Com-3). In most communities, poor financial strength was found as the challenge to continuous financial contributions towards the purchase of medicines in the absence of free delivery from the District Health Directorate (DHDs).

### The act of leadership and partnership in the community health program

How community organizes, coordinates and collaborates with the professional health workers in the program. Findings indicated that Village Health Committees (VHCs) and mother-support groups were the main embodiment of the functioning of the Community Health Actors. The strength of the partnership between health actors and health professionals was generally rated as good, however, the functioning of Village Health Committees (VHCs) and community structures involved in coordinating and managing iCCM were almost not existing in 80% of the communities. For instance, few Community Health Actors (CHAs) had explicitly disclosed that they held meetings with local health professionals to discuss issues concerning iCCM. A critical factor cited as a hindrance to this engagement was the non-existence of the Village Health Committees and mother support groups in the communities.

However, further discussions with health professionals reaffirmed VHCs had existed as part of the national iCCM initiative to support health delivery activities. Nevertheless, their activeness has been weak and less functional in most communities. Unfortunately, a long period of no training, inability to organize and engage VHC have contributed to their dormancy. As indicated, *“Yes, we have the VHCs in the communities, but they are non-functional, their formation has been weak for years and we may need to re-establish them if we have means…funds”* (Female, Health professional). However, in some communities, CHAs disclosed that their collaboration and partnership with the CHOs fostered cordiality and enables them to jointly discuss local health issues with the professionals. Invariably, a strong system of local leaderships and active participation can trigger the change in local situations. Therefore, it is beneficial to consider the locals’ push approach to sustainability, where locals’ capacities are built to drive and sustain the process.

## Discussions

Although the health state of the hard-to-reach communities warrants the iCCM program, a holistic community needs assessment to create awareness, foster a common understanding of the health situation for acceptance and ownership of the program was invisible (see Fig. 1). The conduct of needs assessment was expert-driven, less systematic amid low participation by the local actors [33]. The initiation of the program took the form of expressed needs and informed by relative needs. Community Health Actors (CHAs) had no opportunity to make inputs on the design other than to accept the program as a priority to their children health needs. The effort to instilling locals’ ownerships through participation remains low [34, 42, 43]. Again, the inward desire of the locals to holistically embrace and seek answers about the program remains immure, amidst likewise marginal efforts of health experts to influence locals’ desires (see Fig. 1).

Nevertheless, the experts-led definition of the program preceded by a participatory selection of Community-Based Health Volunteers (CBHVs) from and within their communities, through collaboratively defined criterion (see Fig. 1). Locals awareness was created on the health program towards utilization [41]. More caregivers participated in the decision-making process in selecting CBHVs. The novelty of this is that the CBHVs are knowledgeable of local health situations and require the basics of passion and collective community support to meet local health needs. Though this remains a novel practice across some African countries, especially in Zimbabwe [44], it differs from earlier observation in rural Malawi where the CBHVs were selected, aliens. Also, unlike rural Malawi, where the selected non-residents frequently absent themselves [45]. The benefits communities derive in this context was the direct interaction and all-weather access to the CBHVs for basic childcare. Nonetheless, much effort is required for collective action, in resource mobilization, because the effort of only the selected CBHVs has a little guarantee of attaining sustainability. Though some CBHVs contributed enormously to support the activities of the program with funds due to low support from community members, their low financial capacities could not permit continuity.

Participation is reduced to locals’ acceptance and an event, devoid of participatory needs assessment and monitoring of progress for sustainability. Most local actors perceived their support in the monitoring of the activities of the CBHVs as technical and a typical act of Community Health professionals. Except in Mantari, evidence of community-driven monitoring was less visible (see Fig. 1). Though some residents expressed their intuitive ways of assessing the CBHVs activities in the community, some key actors, expressed a lack of knowledge in the specific activities to monitor. This might have been shaped by the low involvement of local actors in the design of monitoring indicators and the implementation of community-based health programs [31, 46]. Experts perceived monitoring as a task beyond locals’ efforts, which inevitably remains a challenge to the holistic participation of the locals. However, in some rural communities, especially in Uganda, the provision of an agreed action plan to guide locals in the monitoring of the activities of the CBHVs culminated in improved functioning of local health services actors in their respective communities [36]. Therefore, it is worth indicating that whiles the shift from experts towards locals’ hands is imperative for local health governance [47], it is paramount, that such efforts create a common ground for joint analysis of the community health challenges and priorities to gain a communal sense and self-support.

Moreover, though the aim was to ensure that CHAs identify and mobilize local networks and resources for iCCM at the community level [24], including financial and non-financial resources to support the purchase of medicines, the outcome of the participation is observable in the limited resource mobilization and the non-willingness of the community-level actors to mobilise materials and financial support for iCCM activities. As shown in Fig. 2, Except in Siiro, where their participation in needs’ assessment, the selection of the Community-based health volunteers, community-driven monitoring, resources mobilisation and the act of leadership was rated high compared with other communities (the discussants rated their overall level of participation as 22). Although local health actors hold some abilities, useful for building partnership with the modern healthcare practitioners as observed by Haruna and others (14), the communities blamed financial incapacity and irregular provision of medicines from the district health centres as factors limiting their zeal to support CBHVs. Whilst earlier studies showcased the enormous capacities of community health actors in the act of advocating and mobilizing support for community programs [41, 48, 49], such capabilities were visible across the communities in the context of local governance but never translated materially and financially in support for the iCCM. This can be attributed to the low involvement of CHAs as found in the case of Mozambique [50–53]. Moving forward, health officials must maintain their momentum for engagement with VHCs and CHAs, with a keen focus on training and empowerment to unlock their capabilities and improve iCCM [53].

## Conclusion

It is difficult to detach professional health officers beyond experts when collaborating with local actors in pursue of community health and welfare. This is demonstrated in the findings of the study where community participation is regarded more or less as an event than a process towards an outcome. The findings suggest the need to expand the concept of community involvement in iCCM to facilitate communities’ participation and contribution to healthcare service delivery. It points to the needs to involve communities in the development of monitoring indicators to foster a collective contribution to health programs, especially in rural communities. It is also paramount that community-health officers assigned to communities are given refresher training, especially on participatory planning tools. Overall, the collaboration between health experts and social scientists in the form of transdisciplinarity is required for engineering and scaling up community-based health programs, especially towards empowering VHCs, CBHVs and CHAs to realize success through “locals” participation.

## Data Availability

The pdf version of the qualitative datasets collected and analysed for the current study is attached. Interested researchers can conduct Francis Dakyaga, an email address; fdakyaga.fd@gmail.com

## Footnotes

1 Wa the capital city of the Upper West Region.
